# Plants partition the pollinator niche by depositing pollen on different parts of the pollinator body

**DOI:** 10.1371/journal.pone.0323577

**Published:** 2025-05-12

**Authors:** William A. Arteaga-Chávez, Catherine H. Graham, Esteban A. Guevara, Boris A. Tinoco

**Affiliations:** 1 Programa de Recursos Naturales Renovables, Universidad del Azuay, Cuenca, Ecuador; 2 Swiss Federal Institute for Forest, Snow and Landscape Research WSL, Zürcherstrasse 111, Birmensdorf, Switzerland; 3 Grupo de Investigación en Ecología y Evolución en los Trópicos-EETrop, Universidad de las Américas, Quito, Ecuador; 4 Conservation Ecology, Department of Biology, Philipps-Universität Marburg, Karl-Von-Frisch-Straße 8, Marburg, Germany; 5 Facultad de Ciencia y Tecnología, Universidad del Azuay, Cuenca, Ecuador; University of Murcia: Universidad de Murcia, SPAIN

## Abstract

Niche partitioning of pollinators promotes the maintenance of high plant diversity in tropical environments. The role of animal pollinators in this partitioning has been evaluated predominantly at individual and species levels. However, pollinators can carry pollen on different parts of their bodies, potentially resulting in an increase in plant niche partitioning. Nonetheless, studies of pollen loads on different body parts of pollinators and how those patterns influence in plant niche partitioning remain scarce. Here, we 1) measure pollinator niche partitioning of plants considering hummingbird body parts, and 2) explore the contribution of hummingbird traits to niche partitioning of plants. We used mist nets to capture hummingbirds in the southern Andes of Ecuador, and took pollen samples from their bill, base of the bill, forehead, throat and chest-belly using fuchsin-gel. We evaluated plant niche partitioning at the species level based on all pollen found on a given species and at the body-part level by considering pollen loads on different hummingbird body parts, using the specialization metric (d’) and beta diversity analysis. Niche partitioning of plants was higher when the different body parts of hummingbirds were considered than specialization at the species level. The contribution to plant niche partitioning by hummingbird species was positively related to tarsus length, potentially because this trait is associated to hummingbird perching behavior and longer contact times with flowers. In sum, we show that plants increase niche partitioning as a result of pollen deposition on different body-parts, which may help explain coexistence in species-rich systems where many plant species co-flower and share pollinators.

## Introduction

Animal pollinators play a key role in the maintenance of plant populations [1–4]; however, the contribution of different pollinators to plant fitness depends on the quantity and quality of visits to flowers [5,6]. Conspecific pollen deposition on flower stigmas is important in terms of the quality of visits for successful pollination [7,8], but pollinators often visit multiple plant species [9,10], and deposit heterospecific pollen, which can decrease the quality of a visit [11]. The heterospecific pollen carried by a pollinator can be reduced if plants deposit pollen on different parts of a pollinator body which can result in increased partitioning of pollinator niches [12–14]. Nonetheless, pollination studies usually focus on the visitation patterns of pollinator species, neglecting the pattern and diversity of pollen loads carried by pollinators on different body parts [15]. Such partitioning of the pollinator niche by plants could help to explain coexistence of plants in species rich systems where many species co-flower and share the same pollinators.

**Fig 1:**
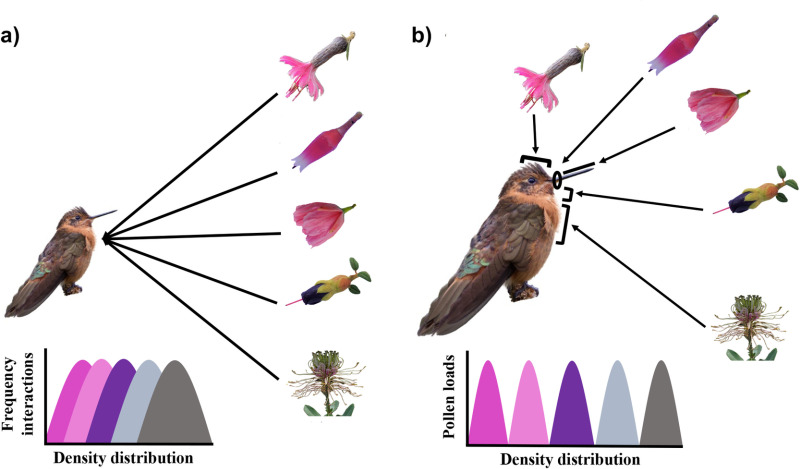
The study of pollen distribution on different body parts of pollinators could provide new insights on how plants partition the pollinator niche. a) Pollinator niche overlap is observed when plant pollination niches are studied using visitation patterns or pollen loads at the species level of the pollinator. b) Pollinator niche partitioning may increase when the different parts of the pollinator body are considered. Photo of the hummingbird *Aglaeactis cupripennis* provided by Gonzalo Nazati.

Niche partitioning of pollinators promotes the coexistence of plant species by 1) reducing competition for pollinators [1,16,17] and 2) increasing the likelihood that enough conspecific pollen for successful pollination is deposited on a flower [7,18–20]. Commonly, the pollinator niche is considered at the species level, where visits of pollinators are used to determine the pollination niche of a plant (e.g., Fig 1a) [21–23]. However, differential patterns of pollen transport have been observed between sexes or individuals of the same pollinator species, suggesting a finer scale of plant pollinator niche partitioning [24–27]. In particular, the bodies of pollinators could contribute to increasing the available pollination niche space for a plant if plant species deposit pollen on different body parts [12–14,26,28–30]. Therefore, the partitioning of the pollination niche should consider these body parts (e.g., Fig 1b) in addition to the common approach of considering the pollinator species or individual as a single pollen transport entity.

Niche partitioning has been studied by using different approaches that measure the exclusivity of interactions between species [17]. Species-level specialization d’ is a metric commonly used in network ecology to measure the specialization of interactions of a species compared to the availability of potential interaction partners [31]. An increase in specialization d’ among coexisting species indicates high specialization and high niche partitioning [31]. In pollination networks, d’ is commonly used to measure plant’s niche partitioning at the species level, and it can also be extended for body parts of a pollinator to measure how plants partition a pollinator’s body for pollen location. However, when d’ is used in plant-pollinator body part networks, it does not allow to identify the pollinator species with the greatest contribution to plant niche partitioning. To address this limitation, beta diversity analysis can be used to measure changes in the composition of pollen among the different body parts of a pollinator [32–34]. In this sense, a pollinator with high pollen beta diversity indicates that each body part carries pollen of different species, and potentially has a high contribution to plant niche partitioning [8,12,13,35,36].

The functional traits of pollinators influence flower visitation patterns and the pollen loads they carry [14,26,37,38]. For example, in hummingbirds, traits such as bill length, body size and tarsus size influence which flowers they visit [39–44]. Long-billed hummingbirds are highly specialized in the floral resources they visit compared to short-billed hummingbirds [41–43]. Therefore, more specialized hummingbirds could carry less mixed pollen loads, as they visit a narrower range of flower species [45,46]. Hummingbird body size can also determine the carried pollen loads. Larger hummingbirds can move pollen over longer distances, potentially transporting a greater amount of heterospecific pollen [47,48]. Moreover, larger hummingbirds offer more space for the pollen to spread out across its body compared to smaller hummingbirds [47,48]. Lastly, hummingbirds with large tarsi tend to perch or cling in the flowers while feeding [49–52]. This behavior implies more time foraging in a flower, which may result in the direct contact of the anthers of a flower with multiple parts of the body such as the throat, chest or belly. However, there are no studies exploring how these morphological traits relate to the diversity and composition of pollen loads that hummingbirds can carry on their bodies.

To evaluate fine-scale niche partitioning of co-flowering plants, we studied the pollen loads of hummingbird assemblages in three shrubby habitats of the Southern Andes of Ecuador. Hummingbirds are important pollinators in the Tropical Andes [53–55], and are part of specialized interactions with plants [56–58]. Owing to the high diversity of hummingbirds and hummingbird pollinated plants they have been a model system to study pollinator niche partitioning [23,42,43,59]. Hummingbirds offer an opportunity to study if plants partition the pollination niche at the level of body parts and if different traits of hummingbird influence their contribution to pollinator niche partitioning of plants [30,60]. In this context, we address the following questions: 1) How does pollen deposition on different hummingbird body parts influence pollination niche partitioning of plants? We expect greater niche partitioning of plants when considering the body parts of pollinators compared to niche partitioning at pollinator species level. 2) How do different hummingbird traits, such as bill length, body size and tarsus length contribute to niche partitioning of plants? We expect that hummingbirds with longer bills will contribute more to plant niche partitioning because they visit fewer flower types than short-billed hummingbirds. Large-bodied hummingbirds will have a higher contribution to plant niche partitioning than smaller-bodied hummingbirds because they have more space available for the placement of pollen. Hummingbirds with larger tarsi will contribute more to plant niche partitioning than species with smaller tarsi because they tend to perch to feed and pollen can be deposited in different parts of the body.

## Materials and methods

### Study site

**Fig 2:**
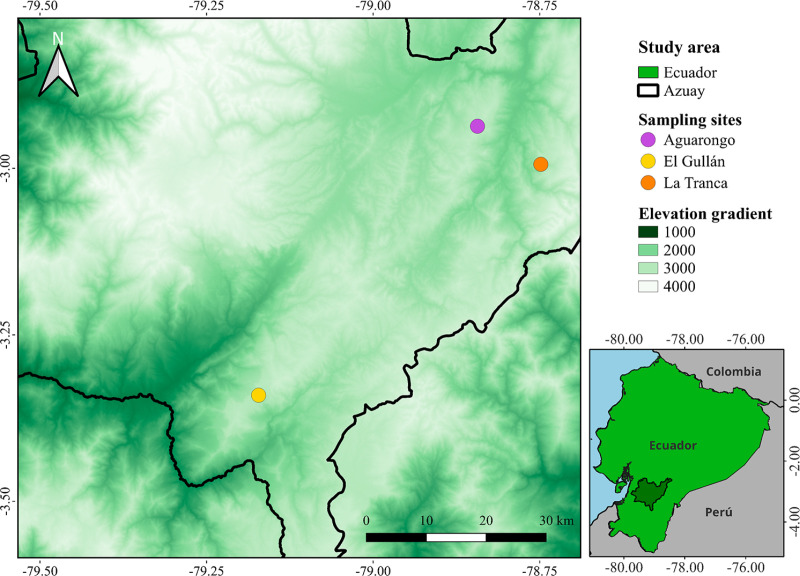
Study sites located in Azuay province in southern Andes of Ecuador. The digital elevation model used for the creation of the figure was obtained from Shuttle Radar Topography Mission (SRTM). Available: https://www.earthdata.nasa.gov/data/instruments/srtm.

We conducted fieldwork in three locations of the southern Andes of Ecuador (Fig 2). The three study sites have shrubby vegetation and had similar elevations. 1) El Gullán is a scientific station, managed by the University of Azuay (-3.340596° S, -79.172850° W, 3,000 m a.s.l). This site is composed of woody shrubland with an irregular canopy of up to 3 m in height. 2) La Tranca is a communal reserve (-2.990075° S; -78.745249° W, 3,040 m a.s.l.) mainly covered by shrubs and some scattered trees that reach up to 4 m in height. 3) El Aguarongo is a protected reserve managed by Consorcio Aguarongo (-2.936617° S, -78.842660° W; 3,160 m a.s.l.) characterized by shrubland and montane forest with an irregular canopy up to 6 m in height. The sites are separated from one another by a minimum distance of 10 km. All three sites have large daily temperature fluctuations between 3 and 18°C. The average annual precipitation ranges from 500 to 1200 mm per/year [61].

### Hummingbird capture and pollen collection

At each site, we established three transects of 300 m, following pre-existing trails, with a minimum distance of 200 m between each transect. The distance between each transect was determined considering topographical and logistical feasibility. We placed a total of 9 transects across the three sites, covering a total of 2700 m in length.

To capture hummingbirds, we placed 10 mist nets along each transect: 5 mist nets of 6 m in length and 5 of 12 m length. The height of all nets was 2.8 m with a mesh hole size of 28 mm. Each site was sampled during three consecutive days, sampling one transect per day from 06h00 to 11h00. The nets were checked regularly at 15 min intervals to minimize the time hummingbirds were trapped in the net and were closed when weather conditions were unfavorable. We conducted three sampling periods at each site, every two months, from August 2022 to February 2023. The average number of species captured at our sampling sites was 6, which is higher than the average of 4.73 species reported in hummingbird studies in the same study area [62,63]. This suggests that our sampling effort was adequate to capture the hummingbird diversity in the area. All captured hummingbirds were identified to the species using the field guide Birds of Ecuador [64].

For each hummingbird captured, we collected pollen samples from five parts of their bodies: 1) bill, 2) base of the bill, 3) forehead, 4) throat and 5) chest-belly (Fig 1b). We selected these body parts because they are the main areas where pollen could be deposited during a visit to a flower [28,29,37]. Pollen samples were taken following the protocol by Stewart & Dudash [12] using a 1 ml plastic syringe containing solidified fuchsin-gel. We used 0.1 ml of fuchsin-gel as the standard quantity to sample each body part. The syringe was pressed until obtaining an exposed section and then we gently rubbed it five times in the same direction or following the pattern of feathers in the sampled body part. All pollen samples were placed in plastic microcentrifuge tubes for transportation. Pollen samples were taken while the hummingbird was in the mist nets to avoid pollen loss due to handling during extraction. Then, hummingbirds were promptly removed and released from the nets to avoid stress.

### Ethics statement

Hummingbirds were captured using mist-nets, handled carefully for data collection, and released immediately at the site of capture. No methods of sacrifice, anesthesia, or analgesia were required, as no invasive or potentially harmful procedures were performed. This study was carried out in strict accordance with the requirements established in the Environmental Unified-Text-of-Secondary-Legislation regarding animal welfare laws, guidelines and policies approved by the Ministry of Environment, Water and Ecological Transition (Ministerio de Ambiente, Agua y Transición Ecológica del Ecuador—MAATE), which authorized scientific research under permit MAATE-ARSFC-2022–2818.

### Hummingbird functional traits

We gathered data on three functional traits that potentially affect pollen load carried by the hummingbird: 1) total bill length, as the length of the bill from base to tip, 2) body mass as the weight of a live individual, and 3) tarsus length, as the length from the outer bend of the tibiotarsal articulation to the base of the toes. We obtained data on all traits measured in male hummingbirds from Tinoco et al. [44]. Some of these functional traits were correlated (body mass - tarsus length, Spearman correlation coefficients r = 0.77, p = 0.04; body mass - bill length r = 0.78, p = 0.04; tarsus length - bill length r = 0.50, p = 0.25). Despite these correlations, each trait is functionally different. Therefore, we run univariate linear models between the traits and our response variable (see Statistical analysis).

### Pollen load counting and identification

The transported pollen samples were fixed in microscope slides in the laboratory. The slides were sampled for pollen grains using a Nikon optical microscope with a 40x lens. On each slide, we drew a 20 x 20 mm square on a plastic acetate sheet and marked 6 verticals and 6 horizontal sampling lines separated by 4 mm. Then we placed the acetate sheet on the cover glass of the slide and counted all the pollen grains that touched any of the 12 sampling lines. Pollen identification was based on a reference collection available in the Herbarium of the University of Azuay. Additionally, we used flower census data collected in the same locations (Bryan G. Rojas unpublished data) to cross-reference pollen identification. We attempted to identify all pollen to species, but since this was not always possible, some were only evaluated to genus. Thus, below we refer to taxonomic entities. To evaluate if the number of sampling lines represented an adequate sample size for pollen, we constructed rarefaction curves using 10 randomly selected slides. The rarefaction curves reached asymptotes in 9 out of 10 slides, indicating our sampling size within a slide captures the potential pollen richness of the whole slide (S1 Fig).

### Niche partitioning of plants using hummingbird pollen loads

We examined whether the pollen loads on different hummingbird body parts increased plant niche partitioning. For this, we compared two types of networks constructed for each study site, combining information from the three transects and three sampling periods. 1) A plant-hummingbird pollen network representing interactions between hummingbird species and plant species (S2 Fig); and 2) a plant-body part pollen network, considering the body parts of each hummingbird species as distinct interaction nodes with plants (S3 Fig). Network links were weighted by the median of pollen grains of each plant species found in each hummingbird or each hummingbird body part of a given species. We explored niche partitioning of plants by using the complementary specialization index d’ at the species level [31]. d’ measures the observed distribution of interactions of a species deviates from a null distribution that assumes random interactions proportional to partner availability [31]. Values range from 0 to 1, values close to 1 indicate high niche partitioning while values close to 0 indicate low niche partitioning.

### Contribution of hummingbirds to plant niche partitioning

To explore how different hummingbird species contribute to pollinator niche partitioning of plants we calculated pollen beta diversity for each captured individual using the equation according to Whittaker [34]. ϒ is the total pollen richness found in the whole body of an individual, and α is the mean pollen richness, calculated from the pollen richness of each body part of that individual. Additionally, we counted the total number of pollen species found on each body part to determine which of the five hummingbird body parts carried the highest pollen richness.

### Statistical analysis

We summarized our findings on captured hummingbird species and pollen counts using measures of central tendency (e.g., mean) and dispersion (e.g., range and standard deviation). Then, we evaluated whether patterns of pollen carried by hummingbird species were independent of the number of individuals captured per species. First, we made rarefaction curves. We used the species of hummingbird with fewest captured individuals as the standardized sample and compared whether the curves representing the pollen patterns of the most captured hummingbird species overlapped with respect to the hummingbird least captured (S4 Fig). Second, we conducted a linear model to determine if the number of captured hummingbirds was related to the carried pollen richness (S5 Fig).

Additionally, we explored the differences in pollen richness on different body parts of hummingbirds using a generalized linear mixed effects model with a Poisson distribution. The dependent variable was pollen richness and the predictor variable was each body part. To consider the potential non-independence of samples obtained from a particular body part, we used the individual from which the sampled was obtained, the species identity and the site where the hummingbird species was captured as random factors. We excluded two species of hummingbirds that were only captured once from the analyses.

### Niche partitioning of plants

We evaluated differences in the niche partitioning (d’) of plant species between the two types of networks, plant-hummingbird and plant-body part, using a linear mixed-effects model. Plant specialization (d’) was the dependent variable and network type was the fixed effect. We used study sites as a random factor to control the lack of independence between samples obtained from the same site and plant species as a cross random effect considering that the same species can occur in multiple sites.

### Hummingbird contributions and the role of their functional traits in plant niche partitioning

Beta diversity of pollen carried in the body of hummingbirds was used as an indicator of the contribution of different hummingbird species to plant niche partitioning. We used a linear mixed effects model to explore the differences in beta diversity among the different hummingbird species, where the dependent variable was the beta diversity index calculated for each individual hummingbird and species identity was the fixed effect. We include the study site as a random factor in the model. Then, we performed a post-hoc pairwise comparison analysis using Tukey’s test to identify pairwise differences between hummingbird species.

We examined how three functional traits of hummingbirds, bill length, body mass and tarsus length, influence the contribution of hummingbird species to niche partitioning of plants. For each trait, we used univariate linear mixed effects models using pollen beta diversity index of hummingbird individuals as the dependent variable and the value of the trait of each species as fixed effects. We used study sites and hummingbird species names as random factors.

All analyses were performed in R [65] using RStudio version 2023.12.1 + 402 [66]. We used the vegan package [67] to build rarefaction curves, bipartite package [68] to build bipartite networks and calculate the specialization index using the function *specieslevel*, the lme4 [69] and lmerTest [70] packages to perform linear models, the car package to obtain a significance value for the models using the function Anova [71], the post-hoc comparisons were done using multcomp package [72], the DHARMa package [73] to evaluate the assumptions of our models (KS test p > 0.05 for all our models) and the ggplot2 [74] to construct plots. In our analyses, we report regression coefficients, p-values as well as marginal (Rm) and conditional (Rc) R-squares. Marginal R-squares takes into account the variance for fixed effects only, whereas conditional R-squares takes into account variance of both fixed and random effects [75].

## Results

**Fig 3:**
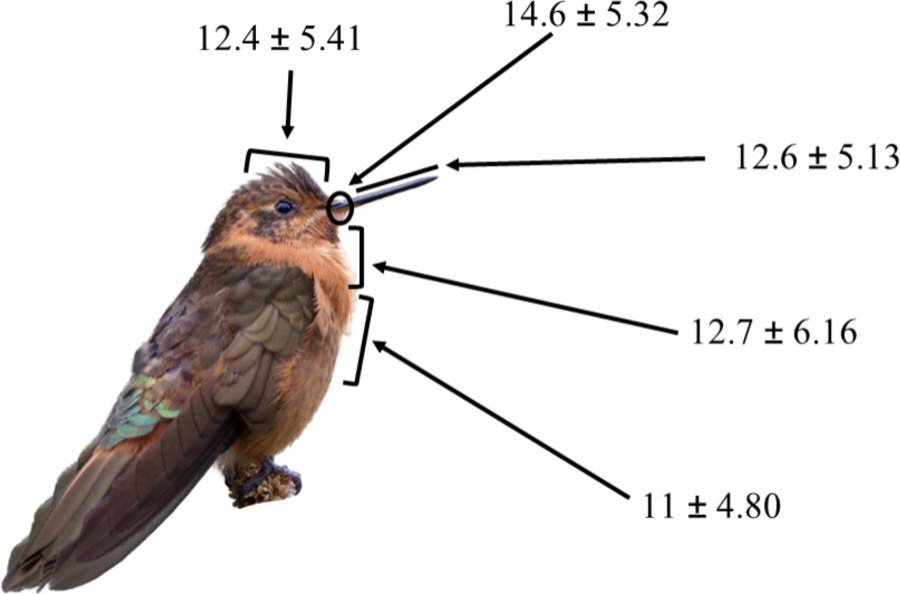
Mean richness of pollen transported by the body parts of hummingbird species from the southern Andes of Ecuador. The results are illustrated showing *A. cupripennis* provided by Gonzalo Nazati.

We captured 145 individuals of 9 hummingbird species during 951.25 net/hours. *Metallura tyrianthina* and *Heliangelus viola* were the species with most individuals captured. Overall, we counted 165,051 pollen grains and identified 37 pollen taxonomic entities. We identified 22 plant species, representing 96.6% of the pollen grains, the remaining 3.3% of the pollen grains was identified to genus level. Taxonomic entities found on the different hummingbird species ranged from 9 to 28, and this richness was not influenced by sample size (S4 Fig and S5 Fig). The total number of pollen grains found in pollen loads carried by the different species ranged from 1,057–101,081 (Table 1). The richness of pollen transported in each body part was different (*χ*2 = 40.34, d.f. = 4, p < 0.001), with the base of the bill as the body part with highest pollen richness (Fig 3).

**Table 1:** Description of pollen loads found in different hummingbird species in the southern tropical Andes of Ecuador.

Hummingbird	Individuals captured	Plant species	Plant species per individual	Total pollen load	Pollen load mean + SD
*Metallura tyrianthina*	57	28	0.49	101,081	1,773.35 ± 2,047.10
*Heliangelus viola*	33	25	0.76	15,382	466.12 ± 518.27
*Coeligena iris*	17	17	1	4,853	285.47 ± 380.85
*Aglaeactis cupripennis*	15	19	1.27	13,437	895.80 ± 1,632.69
*Lesbia nuna*	10	22	2.20	14,352	1,435.20 ± 1,788.18
*Eriocnemis vestita*	7	9	1.29	8,631	1,233 ± 1,176.79
*Lesbia victoriae*	4	11	2.75	4,320	1,080 ± 1,261.43
*Colibri coruscans*	1	11	–	1,938	–
*Lafresnaya lafresnayi*	1	10	–	1,057	–

### Niche partitioning of plants using hummingbird pollen loads

**Fig 4:**
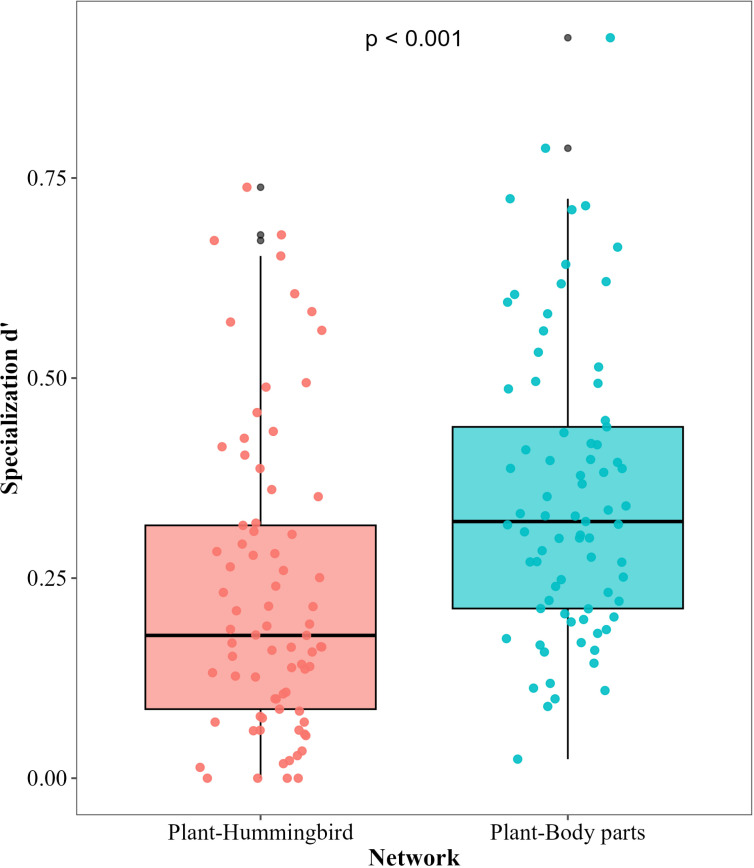
Plant niche partitioning calculated at the level of hummingbird species and at the level of different body parts of hummingbirds. Niche partitioning was measured using pollen loads obtained from hummingbirds in the southern Andes of Ecuador.

We found that niche partitioning (specialization index d’) of plants was higher in networks that considered the different body parts of hummingbirds than those built at the level of pollinator species (*χ*2 = 19.84, d.f. = 1, p < 0.001) (Fig 4).

### Hummingbird contributions and the role of their functional traits in plant niche partitioning

**Fig 5:**
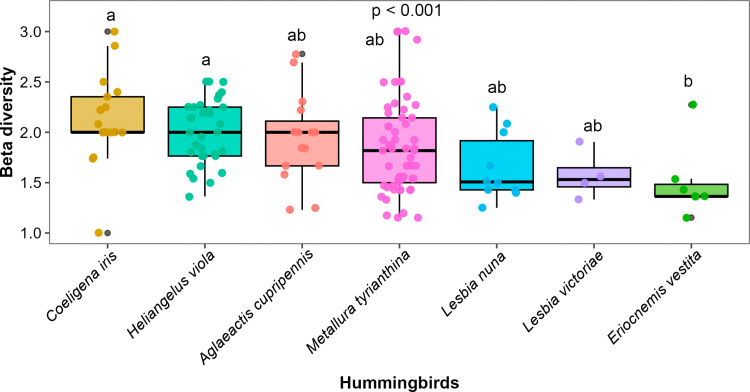
Beta diversity of pollen carried by hummingbirds from the southern Andes of Ecuador. All boxplots show the median as a horizontal line, boxes indicate the 25th and 75th percentiles, whiskers indicate the range of data, and black points are outliers. Different letters indicate statistically significant differences among species.

The contribution of hummingbirds to plant pollination niche partitioning, measured as pollen beta diversity carried by different individuals, was significantly different among species (*χ*2 = 22.04, d.f. = 6, p = 0.001). *Coeligena iris* and *Heliangelus viola* carried the highest beta diversity of pollen loads, while *Eriocnemis vestita* had the lowest. Additionally, these three species showed significant differences in the beta diversity of the pollen they carried (Fig 5).

**Fig 6:**
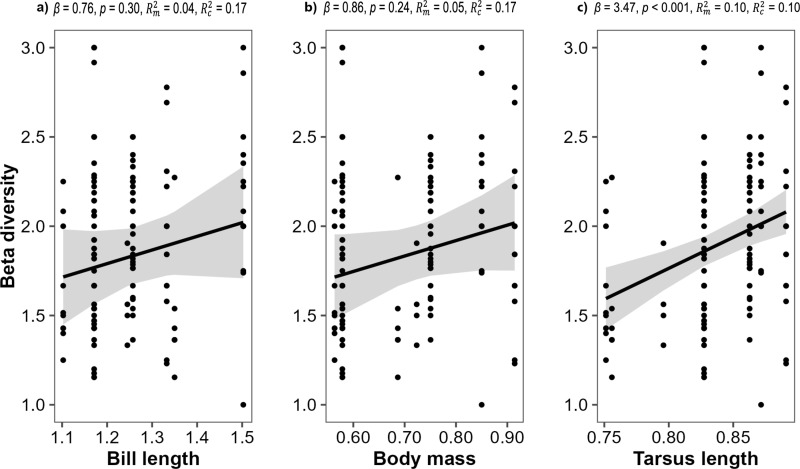
Relationships between a) bill length, b) body mass and c) tarsus length and the beta diversity of pollen loads carried by hummingbirds in the southern Andes of Ecuador. The plots show the observed values of beta diversity carried by each individual captured (n = 143), and the regression line with standard error based on the predicted values of a linear mixed effects model. The functional traits of the hummingbirds were scaled to log10.

Only one functional trait of hummingbirds was associated with the beta diversity of pollen found in hummingbirds. Tarsus length was positively associated with beta diversity of pollen loads, whereas body mass and bill length showed no associations (Fig 6).

## Discussion

The analysis of niche partitioning of plants considering body parts of pollinators has received limited attention in ecology [15,76]. Our findings suggest that plant species deposit their pollen on different parts of the body of hummingbirds, thus increasing their niche partitioning and reducing the overlap in pollinators with other co-flowering plants. Furthermore, we found differences in the contribution of hummingbird species to plant niche partitioning. In particular, hummingbird species with longer tarsi had a high contribution to niche partitioning as indicated by their high levels of beta diversity of pollen loads carried in their body parts. Our study highlights the value of studying pollinator niche partitioning at finer scales in mutualistic pollination systems. Niche partitioning across body parts is overlooked but may be an important mechanism explaining the maintenance of high diversity in co flowering species inhabiting tropical systems.

As we predicted, niche partitioning among plants significantly increases when different body parts of hummingbirds are considered as part of a plant’s niche space. This partitioning in pollen deposition could reduce interspecific competition among plants and promote the coexistence of flowering species [1,17,77]. This mechanism of pollen deposition could not only directly benefit the female fitness of the plant, but also the male fitness because it could prevent pollen from being displaced or buried by pollen of other species on the body of the pollinator [7,11] Several studies have shown high levels of pollinator niche overlap among flowering plants visited by hummingbirds, suggesting potential competition and reduced fitness among co-flowering species [25,43,78,79]. However, these studies are based on visitation data. Our results help to reconcile these findings by showing that plants can have high levels of pollinator niche partitioning by placing pollen in different body parts of a hummingbird. Therefore, while a pollinator can visit and take pollen of different plant species, by transporting pollen of specific species in different body parts, it can still deposit conspecific pollen in the stigma of a flower. Our results add to the growing literature on pollination ecology, highlighting that there are additional dimensions to the species level niche partitioning that likely influence plant coexistence [24–27].

Pollinator species can have different contributions to plant niche partitioning, as shown by our beta diversity analysis of pollen loads across the body parts of hummingbirds. Moreover, we found that tarsus length positively influences pollen beta diversity. In the high Andes, where environmental conditions are windy, hummingbirds tend to have large tarsi, which may facilitate perching and clinging to flowers [50–52]. Our study sites are located above 3000 m a.s.l., and many of the hummingbird species studied belong to the Andean clades of Coquettes (e.g., *H. viola*, *M. tyrianthina*) and Brilliants (e.g., *A. cupripennis*), where several species show the behavior of perching while feeding [50–52]. This feeding behavior likely increases the time hummingbirds spend manipulating a flower [50,52,80], which could allow the male reproductive organs of the flower –anthers– to contact specific body parts of hummingbirds. Thus, the increased probabilities of depositing pollen in specific body parts of a hummingbird while perching from a flower, may explain the high pollen beta diversity carried by hummingbirds with longer tarsi. Future studies should assess this hypothesis with behavioral observations and explore whether tarsus length also influences pollen beta diversity in pollinators across different elevations [15].

Hummingbirds with larger tarsi that carry high pollen beta diversity may deliver pollen more efficiently to the flowers they visit. A high beta diversity of pollen load in a pollinator increases the chances of delivering conspecific pollen to the flowers it visits [28]. Nonetheless, it has been found that land-use change negatively influences the presence of hummingbirds with longer tarsi [81–83]; therefore the loss of native forests could diminish pollination services provided by perching hummingbirds, which rely on flowers with suitable structures for perching and feeding [49,50,52,84]. However, it is necessary to further explore this relationship to understand the link between hummingbird perching behavior and the architecture of flowers that results in pollen being deposited in different body parts. Additionally, it is important to explore the association between pollinator performance and beta diversity of pollen loads in a pollinator [15,25].

The base of the bill of hummingbirds had the highest pollen richness. This is consistent with other studies that have found similar results [29,30]. Interactions between hummingbirds and plants are determined by linkage rules such as the degree of trait-matching between the bill and the floral tube [15,41,42]. Trait–matching may enable the base of the bill to come into contact with the pollen-presenting structures –anthers– of the flower, as these stamens are usually located at the external portion of the floral tube (e.g., *Barnadesia arborea*, *Fuschia* sp., *Bomarea multiflora* and *Tristerix longebracteatus*) [46,85]. Thus, trait matching might explain why the base of the bill is the structure where hummingbirds may carry the highest diversity of pollen loads.

Analysis of pollen loads allowed us to identify the plant species visited by pollinators and determine the location of pollen in their bodies. However, identifying pollen is a challenging task in areas of high plant diversity where reference libraries are not widely available. In our study, however, resolution was not a limitation, as 96.7% of the pollen grains carried by hummingbirds were identified at some taxonomic level. Therefore, we believe that the interactions presented for our study system are representative. Nonetheless, we offer a few caveats to our study. Our sampling captures the phenological variation of flowers over half a year, so we emphasize the importance of conducting research over longer periods, as flowers may seasonally vary throughout the year [43]. We also highlight the importance of analyzing pollen loads in body parts taking into account the sexual identity of hummingbirds, as feeding niches may vary between males and females [27]. Lastly, we cannot disregard the potential influence of network size in our finding of an increase in niche partitioning of plants when the body parts of a pollinator are considered, as there is a positive relationship between niche partitioning and network size [31,56,86,87]. However, we believe that the pollen loads sampled on the captured hummingbirds accurately reflects the real biological process of pollen placement by plants on their pollinators.

Our study shows that plants can deposit pollen on different parts of the body of pollinators, increasing niche partitioning. This observation could help to explain coexistence in species-rich systems, such as the tropical Andes, where plant species may co-flower and share multiple pollinators. We also show that particular functional traits (e.g., tarsus length in hummingbirds) could influence a pollinator’s contribution to niche partitioning, the quality of pollen delivery and ultimately the reproductive success of plants.

## Supporting information

S1 Fig Rarefaction curves of pollen samples taken from hummingbird body parts. (TIF)

S2 Fig Plant-hummingbird pollen network, represents interactions between hummingbird species and different plant species. (TIF)

S3 Fig Plant-body part pollen network, we considered the body parts of each hummingbird species as distinct interactions nodes with the plants. (TIF)

S4 Fig Rarefaction curves of pollen loads carried by each hummingbird species. (TIF)

S5 Fig Linear regression of the effects of the number of captured hummingbirds on the average richness of pollen carried by different hummingbird species. (TIF)

S1 Dataset Pollen loads carried by hummingbirds. (CSV)
