# Supplementary figures and images for: Plants partition the pollinator niche by depositing pollen on different parts of the pollinator body
Source: PLoS One. 2025 May 12;20(5):e0323577. doi: 10.1371/journal.pone.0323577 (PMC12068658; doi:10.1371/journal.pone.0323577)

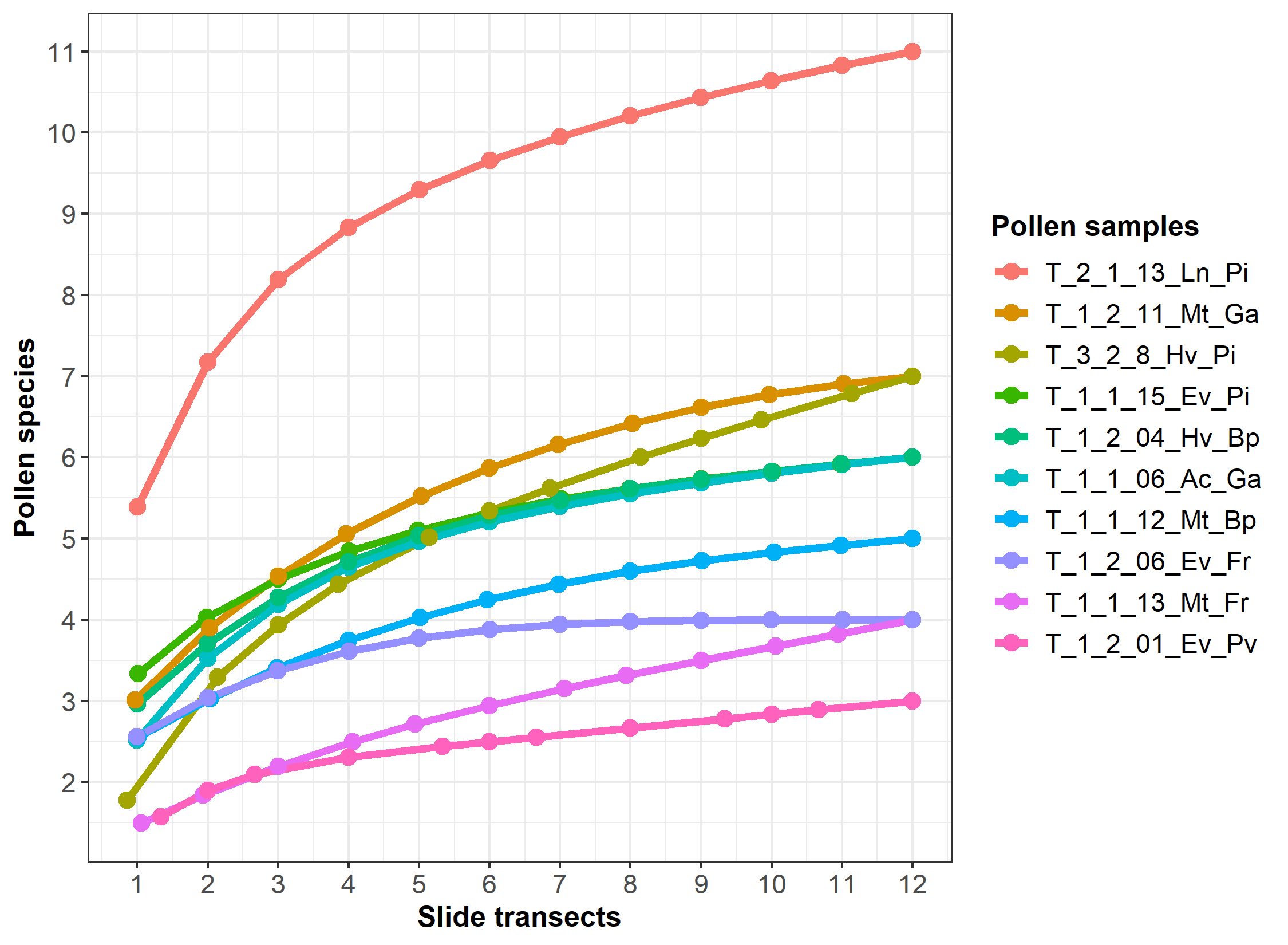

Supplement: S1 Fig — (TIF) [file pone.0323577.s001.tif]

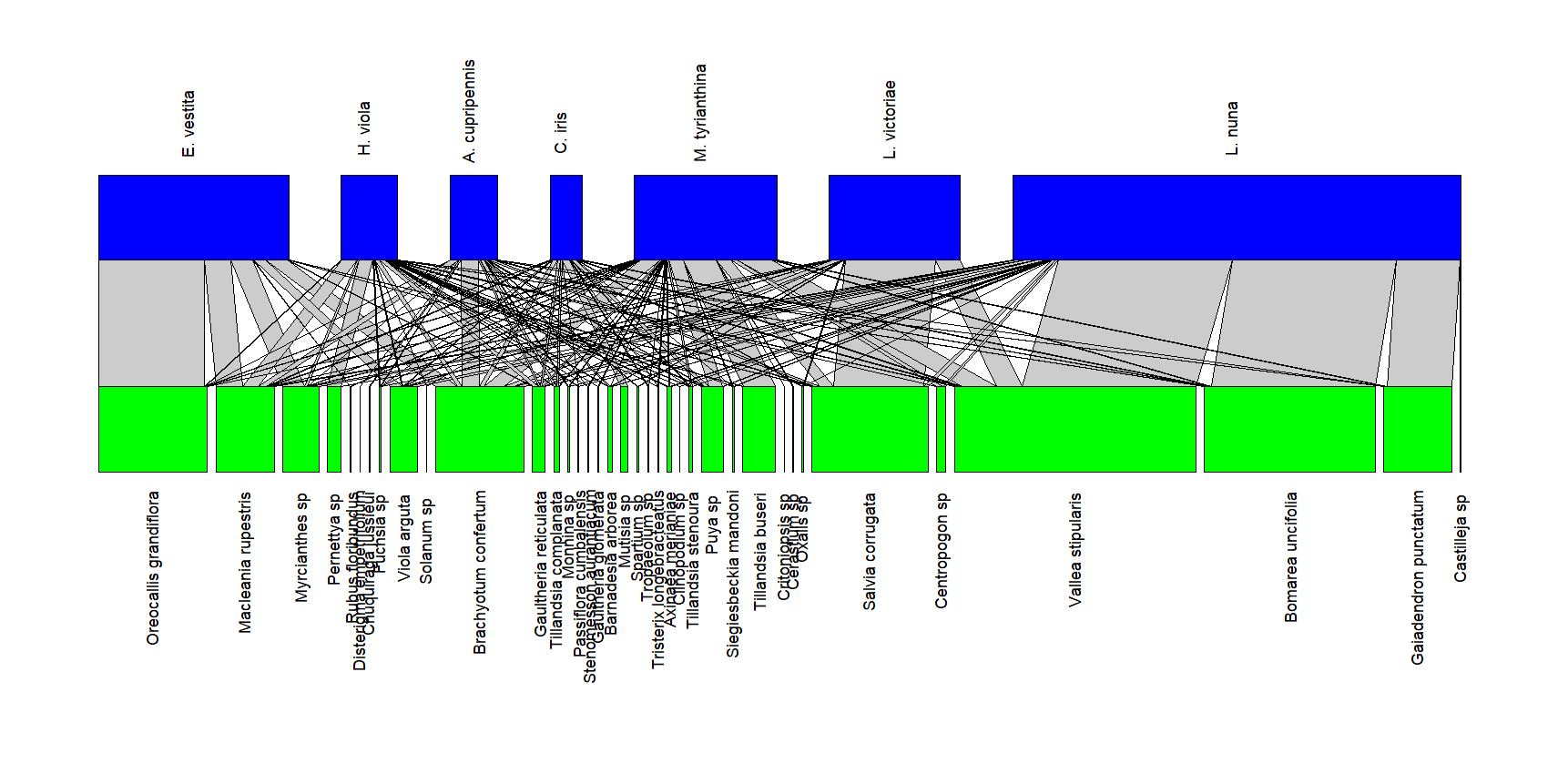

Supplement: S2 Fig — (TIF) [file pone.0323577.s002.tif]

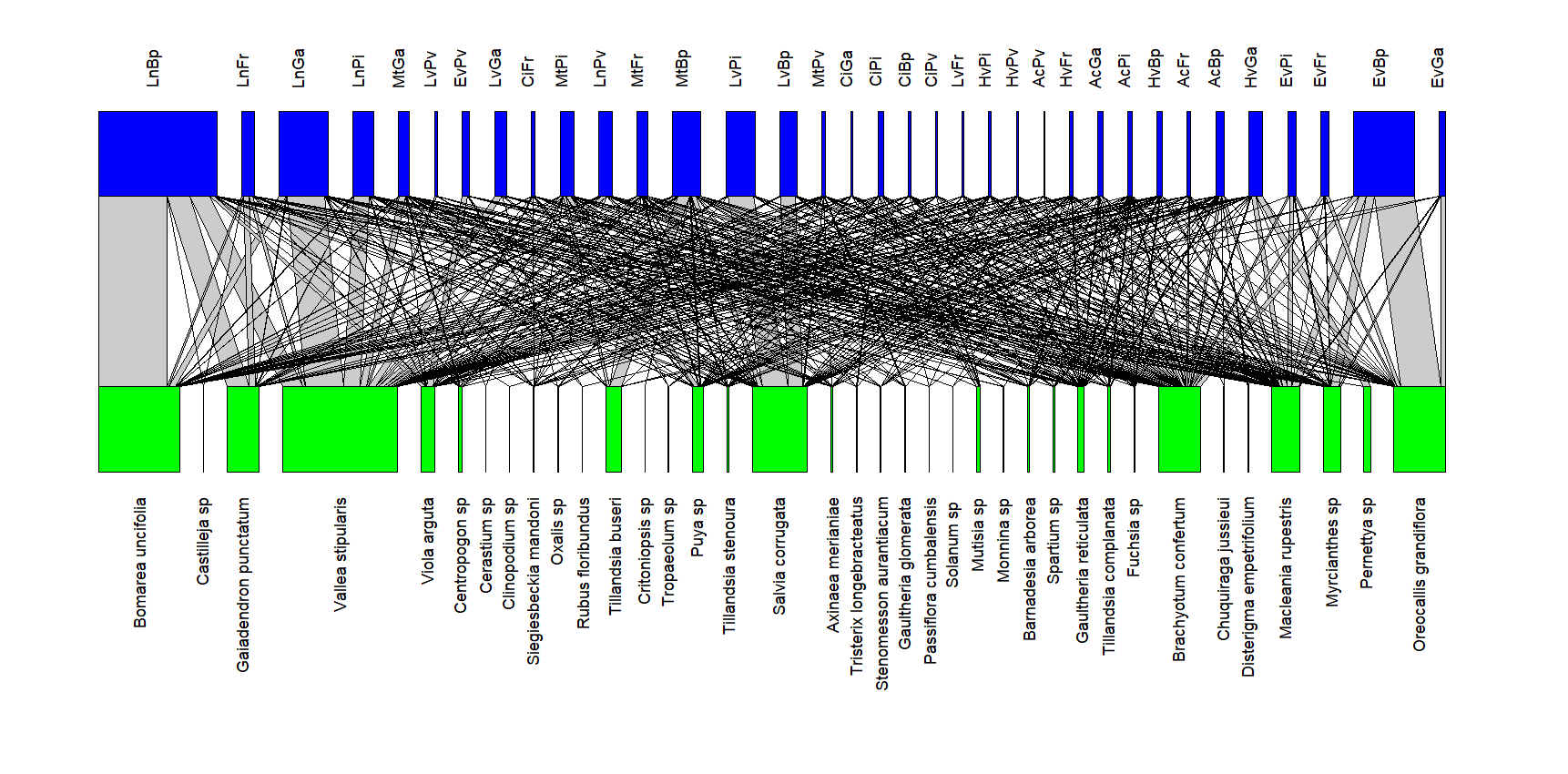

Supplement: S3 Fig — (TIF) [file pone.0323577.s003.tif]

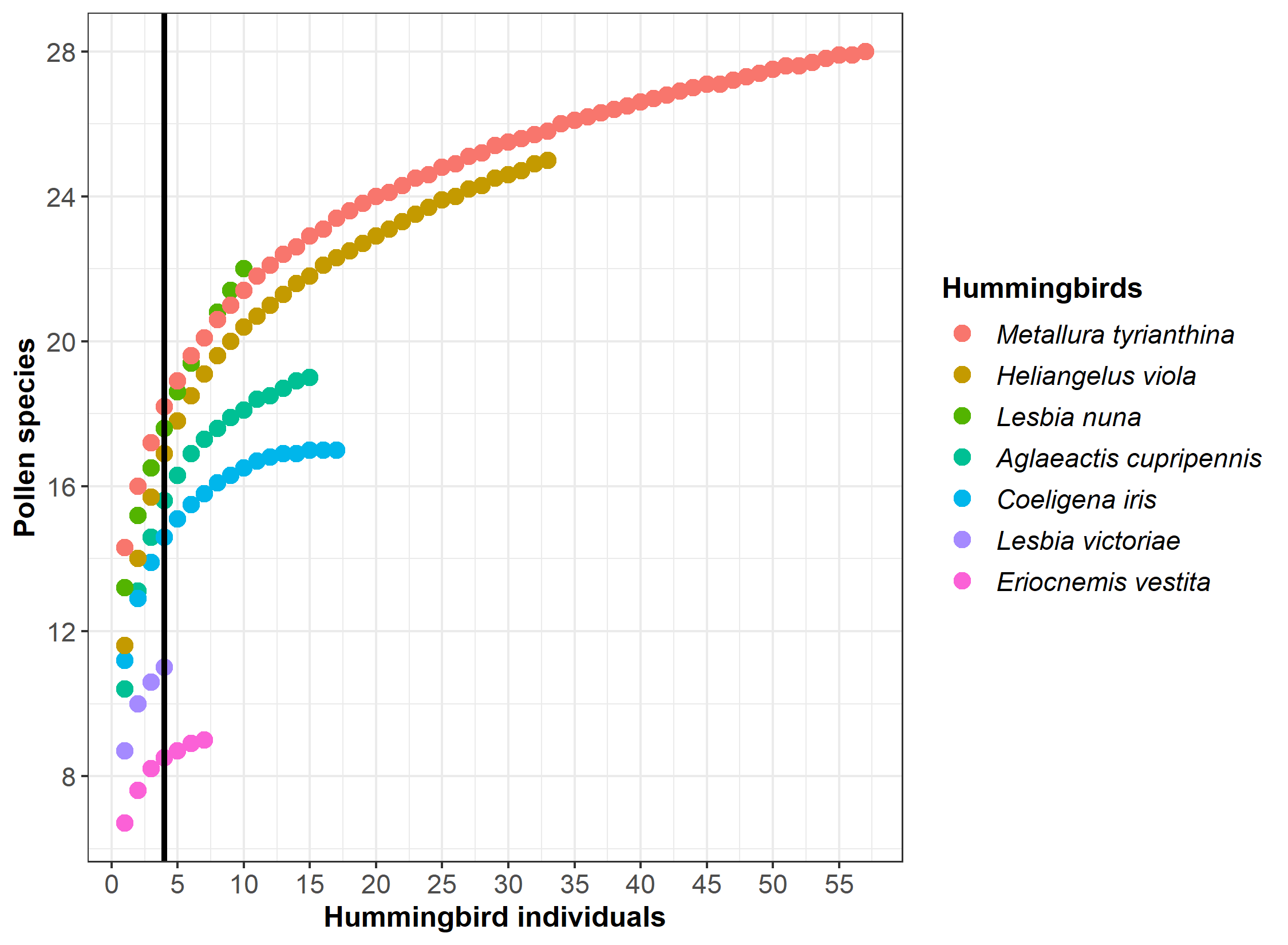

Supplement: S4 Fig — (TIF) [file pone.0323577.s004.tif]

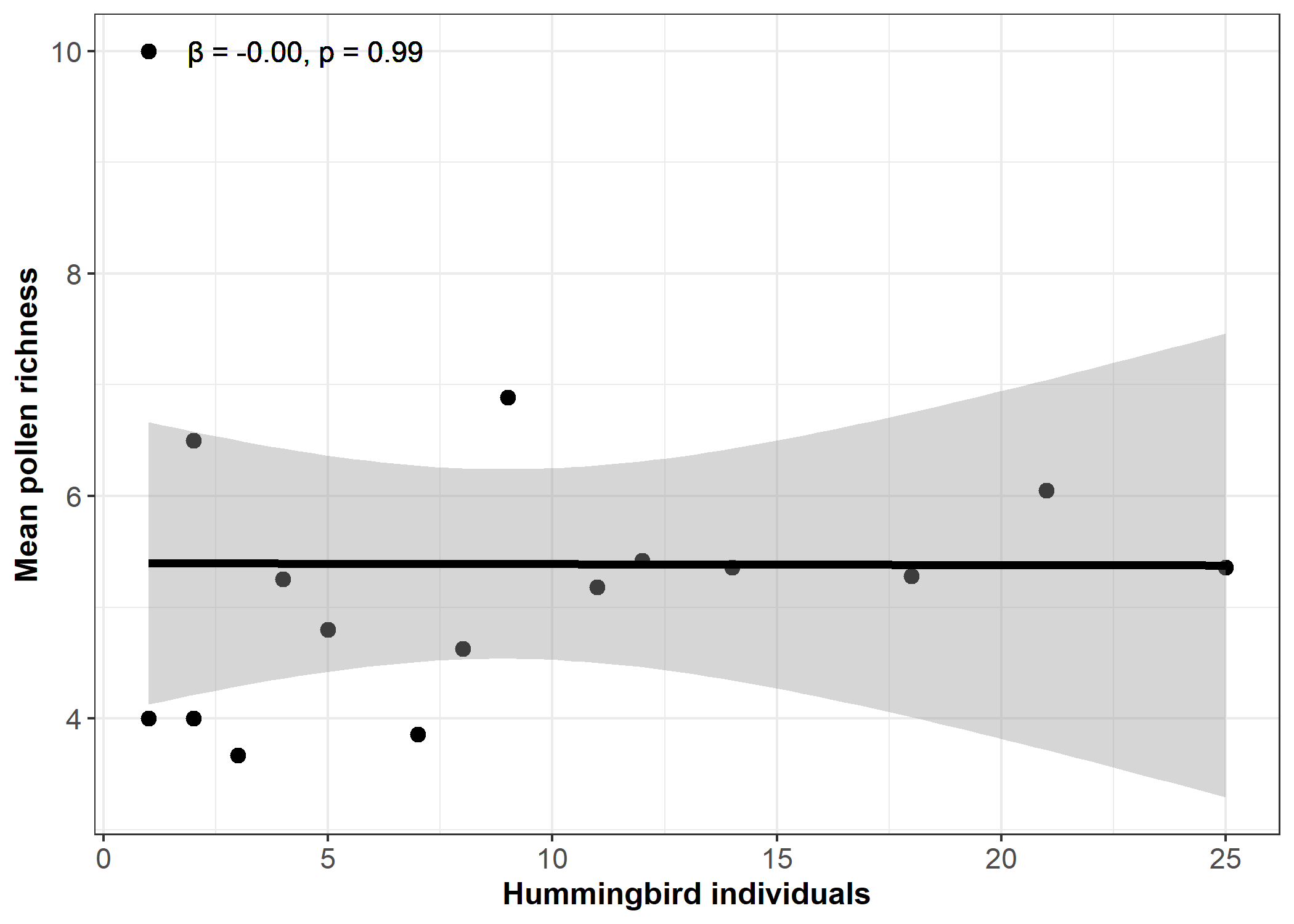

Supplement: S5 Fig — (TIF) [file pone.0323577.s005.tif]
